# Central nervous system histoplasmosis: Multicenter retrospective study on clinical features, diagnostic approach and outcome of treatment: Erratum

**DOI:** 10.1097/MD.0000000000010537

**Published:** 2018-04-20

In the article, “Central nervous system histoplasmosis: Multicenter retrospective study on clinical features, diagnostic approach and outcome of treatment”,^[1]^ which appeared in Volume 97, Issue 13 of *Medicine*, Dr. Holenarasipur R. Vikram's middle initial was left out.
